# Enhancement of Thermoelectric Performance by Reducing Phonon Thermal Conductance in Multiple Core-shell Nanowires

**DOI:** 10.1038/srep07150

**Published:** 2014-11-21

**Authors:** Wu-Xing Zhou, Ke-Qiu Chen

**Affiliations:** 1 Department of Applied Physics, Hunan University, Changsha 410082, China

## Abstract

The thermoelectric properties of multiple core-shell nanowires are investigated by using nonequilibrium Green's function method and molecular dynamics simulations. The results show that the thermoelectric performance of multiple core-shell NWs can be improved observably with the increase of shell number compared with the single component NWs due to the significant reduction of phonon thermal conductance. The ZT value of multiple core-shell NWs can reach three times greater than that of the single component GaSb NWs at room temperature. Moreover, the ZT values of both the core-shell NWs and single component NWs are increased with the increasing temperature, but the ZT value of core-shell NWs increases more slowly than that of single component NWs. These results show that the single component NWs is suitable as thermoelectric material at much high temperature, but the multiple core-shell NWs is more suitable as thermoelectric material at room temperature.

The thermoelectric energy conversion technology, which can directly convert heat into electricity and vice versa without moving parts, have received considerable attention in recent years due to their great potential in both cooling system and power generation^1^,^2^. The most important factor that determines the conversion efficiency is captured by a dimensionless figure of merit of the materials, defined as *ZT* = *σS*^2^*T*/(*λ_e_* + *λ_ph_*), where *σ* is the electrical conductivity, *S* is the Seebeck coefficient, *T* is the absolute temperature, *λ_e_* is the electrical thermal conductivity, and *λ_ph_* is the phonon thermal conductivity. A good thermoelectric material has a large ZT value which requires a large power factor (*σS*^2^) along with a low thermal conductivity (*λ_e_* + *λ_ph_*). However, it is very difficult to significantly improve the thermoelectric performance of conventional materials because the *σ*, *S* and *λ_e_* are coupled unfavorably^3^,^4^. For instance, a high electrical conductivity usually leads to a low Seebeck coefficient and a high electrical thermal conductivity, both of which are undesirable for thermoelectric applications. A major breakthrough came in the 1993, Hicks et al. theoretically predicated that low-dimensional structures may increase the *ZT* value by reducing lattice thermal conductivity with phonon-boundary scattering^5^. Since then, a strong research activity has been focused on the low-dimensional structures, such as superlattices^6^,^7^, nanowires (NWs)^8^,^9^,^10^,^11^,^12^, nanoribbons^13^,^14^,^15^, nanocomposites^16^,^17^, etc, for thermoelectric applications.

Recent years, semiconductor NWs have received considerable attention due to their great potential for thermoelectric application^18^. Primary interest in nanowires is motivated by the significantly suppressed phonon thermal conductivity due to the phonon-boundary scattering and possible phononics engineering^19^,^20^,^21^. Another advantage of nanowires is a high power factor due to enhanced electrical density of states near the Fermi level, which is caused by quantum confinement^5^. Indeed, recent experimental and theoretical efforts have demonstrated that silicon NWs can be regarded as efficient thermoelectric materials although bulk silicon is not^22^. In addition, compared to the single component semiconductor NWs, the physical properties of core-shell NWs can be further manipulated by changing the configurations and compositions. For example, intrinsic core-shell NWs exhibit high carrier mobility due to the suppression of ionized impurity scattering and surface charges^23^. At the same time, the core-shell NWs have a very low phonon thermal conductance due to the enhancement of surface scattering^20^. So the high mobility and low phonon thermal conductance may give us an opportunity to improve the thermoelectric properties of the NWs.

However, there are only a few theoretical studies on the thermoelectric properties of Si/Ge core-shell NWs^24^,^25^,^26^, and the thermoelectric properties in multiple core-shell NWs are still in its infancy. Recently, GaSb/InAs and InAs/GaSb core-shell NWs have been fabricated experimentally, and exhibit excellent electrical performances^27^. Therefore, in the present work, we investigate systematically the thermoelectric properties of GaSb/InAs and InAs/GaSb core-shell NWs. To further improve the thermoelectric performance, we also research the thermoelectric properties of multiple GaSb/InAs core-shell NWs. The results show that the thermoelectric performance of both the GaSb/InAs core-shell NWs and InAs/GaSb core-shell NWs are improved observably compared with the single component NWs. We also find that the thermoelectric performance of multiple core-shell NWs can be further improved significantly with the increase of shell number. The *ZT* value can reach three times greater than that of the single component GaSb NWs at room temperature. Moreover, we also investigate the influence of temperature on the thermoelectric properties in core-shell NWs. The results show that the *ZT* values in core-shell NWs and single component NWs are both increased with the increasing temperature. However, the *ZT* value in core-shell NWs increases more slowly than that of single component NWs, which means the multiple core-shell NWs is more suitable as thermoelectric material at room temperature.

## Results

**Figure 1:**
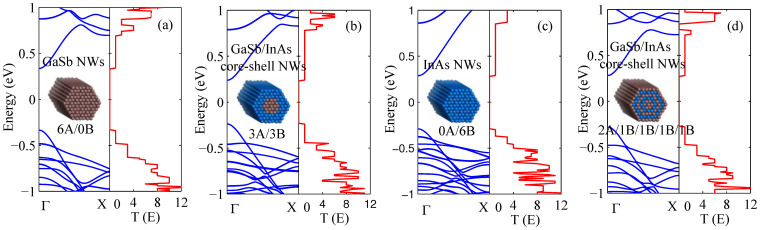
Energy band structure and electron transmission function for (a) GaSb NWs, (b) GaSb/InAs core-shell NWs (3A/3B), (c) InAs NWs, (d) GaSb/InAs core-shell NWs (2A/1B/1B/1B/1B). The inset is the corresponding geometry.

**Figure 2:**
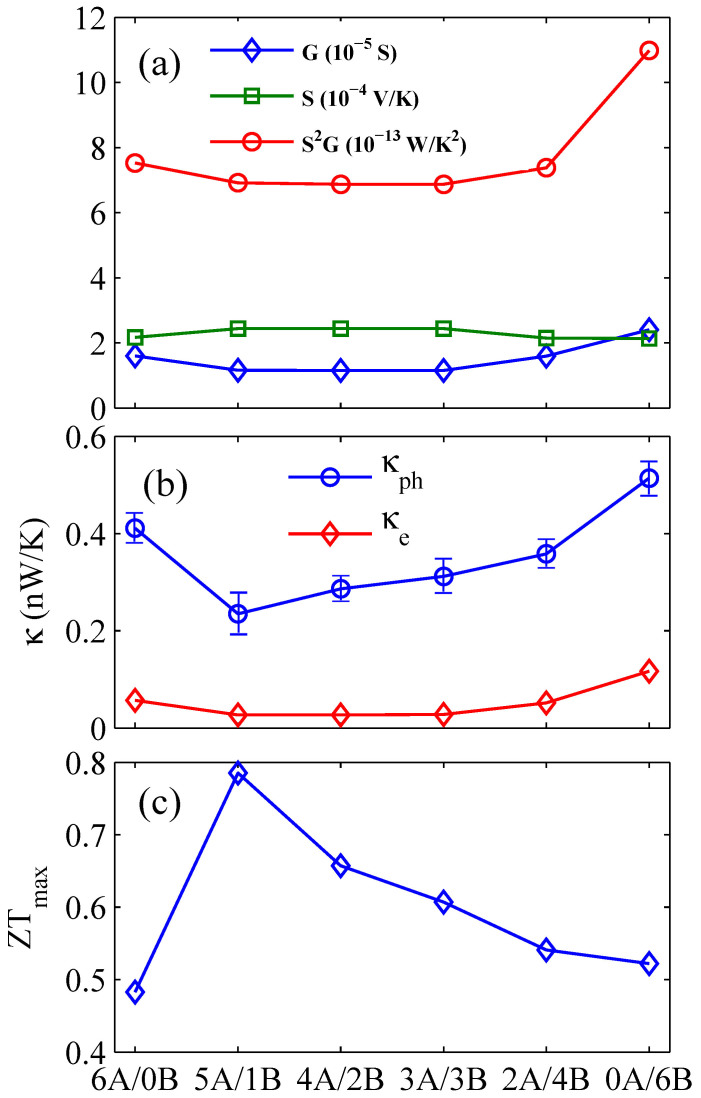
(a) Electrical conductance *G*, Seebeck coefficient *S*, and power factor *P*, (b) Phonon thermal conductance *κ_ph_* and electron thermal conductance *κ_e_*, (c) The optimal *ZT* of GaSb/InAs core-shell NWs with different proportions at room temperature, in comparison with those of GaSb NWs and InAs NWs.

To research the influence of core-shell structure on the electron transport properties, we plot the energy band structure and the electrical transmission function for GaSb NWs, GaSb/InAs core-shell NWs, InAs NWs and GaSb/InAs multiple core-shell NWs, as shown in Fig. 1(a)–(d), the inset is the corresponding geometry. Here, we adopt the notation *mA/nB* to designate the GaSb/InAs core-shell NWs. *A* and *B* indicate the GaSb and InAs, *m* and *n* indicate the radius of core and the thickness of shell respectively. For instance, the 4*A*/2*B* indicates that the radius of GaSb core is of four layers atoms; the thickness of InAs shell is of two layers atoms. From Fig. 1, it is clearly shown that the transmission functions display clear stepwise structures, which shows the number of electron channels. The quantized transmission can also be obtained by counting the numbers of energy bands at any given energy. Based on the electron transmission function, we calculate the electrical conductance *G*, the Seebeck coefficient *S*, and the power factor *S*^2^*G* of GaSb NWs, GaSb/InAs core-shell NWs and InAs NWs at toom temperature, as shown in Fig. 2(a). It is worth saying explicitly that we use the electrical conductance to define the power factor instead of electrical conductivity, because the electrical conductivity in a nanoscale system cannot be well defined since the cross-sectional area is not well defined, and the *ZT* will not be affected because we use the thermal conductance in the definition of *ZT*, not thermal conductivity at the same time. Here, the *G*, *S* and *S*^2^*G* correspond to the optimal values to achieve the maximum value of *ZT*. It is shown that the core-shell NW has a lower electrical conductance compared with the single component NW, but the Seebeck coefficient of core-shell NW is higher than that of single component NW. In sum, the power factor is little changed in different core-shell NW.

To evaluate the *ZT* value explicitly, we calculated the phonon thermal conductance and electron thermal conductance of GaSb/InAs core-shell NW with different proportions as shown in Fig. 2(b). The electron thermal conductance corresponds to the optimal value to achieve the maximum value of *ZT*. It is shown that the phonon thermal conductance is one order of magnitude higher than electron thermal conductance, which shows phonon thermal conductance being dominant in semiconductor NW. This figure also shows that the phonon thermal conductance decreases at first and reaches a minimum value at 5*A*/1*B*, and then increases with the further increase of the thickness of shell. The similar trend also appears in InAs/GaAs core-shell NWs and Si/Ge core-shell NWs^20^,^28^. The reduction in the thermal conductance of core-shell NWs stems from the depression and localization of long-wavelength phonon modes at the core-shell interface and high frequency nonpropagating diffusive modes^20^, and the increase in thermal conductance with the increase of shell thickness is because the dominant heat transfer channel changes from core to shell^28^. In addition, it is worth noting that the electrical conductance has a similar trend with electron thermal conductance, since the charge carriers are also heat carriers. Actually, the correlation between electrical conductance and electrical thermal conductance is one of the main reasons that most of the experiments by regulating the carrier concentration failed to achieve high *ZT*.

Based on the calculated power factor *S*^2^*G*, the electrical thermal conductance *κ_e_*, and the phonon thermal conductance *κ_ph_*, we calculate the maximum *ZT* value of core-shell NWs with different proportions at room temperature as shown in Fig. 2(c). The *ZT_max_* curve first increases and reaches the maximum value at 5*A*/1*B*, and then decreases with the further increases of the shell thickness. The optimal *ZT* value can reach 0.79, which is about 65% higher than that of single component GaSb NW. It is because the 5*A*/1*B* has the lowest thermal conductance, at the same time, the power factor is not deteriorated. In other words, the lower thermal conductance combining with the unaffected power factor produces the peak *ZT*.

**Figure 3:**
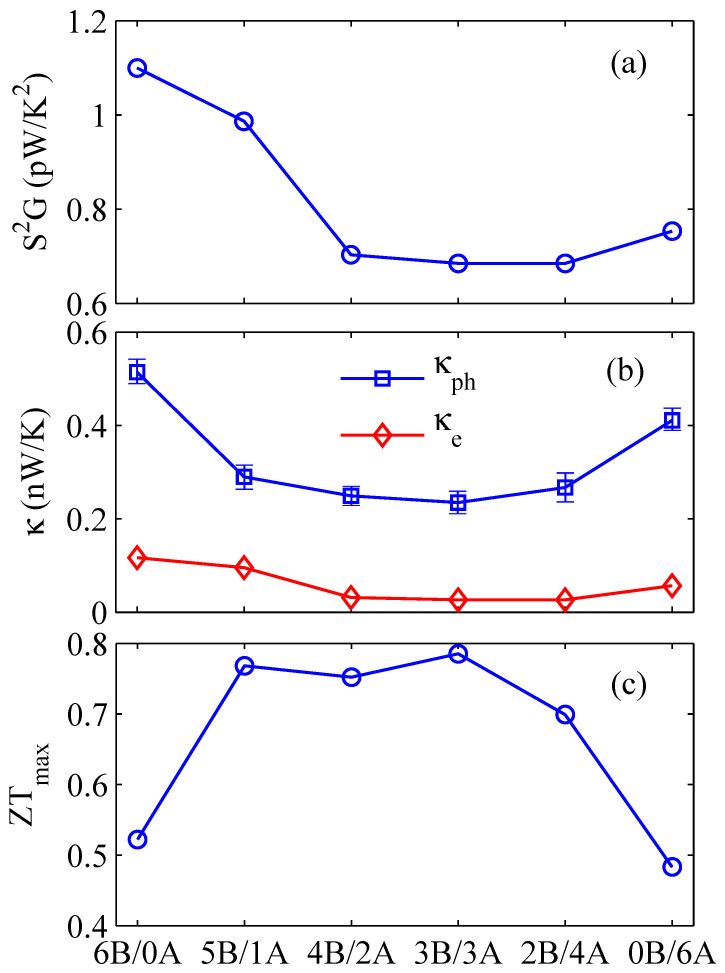
(a) Power factor *P*, (b) phonon thermal conductance *κ_ph_* and electron thermal conductance *κ_e_*, and (c) the optimal *ZT* of InAs/GaSb core-shell NWs with different proportions at room temperature, in comparison with those of InAs NWs and GaSb NWs.

To prove the conclusion that the thermoelectric performance of core-shell NW is much better than that of single component NW is universal, we also calculated the thermoelectric properties of InAs/GaSb core-shell NW at room temperature as shown in Figs. 3(a)–(c). Fig. 3(a) shows the optimal power factor in different proportions InAs/GaSb core-shell NW. It is clearly shown that the power factor in core-shell NW is lower than that in single component InAs NW. The similar trend also appears in the electrical thermal conductance due to the Wiedemann-Franz law as shown in Fig. 3(b). Besides, the phonon thermal conductance in InAs/GaSb core-shell NW rapidly goes down compared with the single component NW. This result is consistent with above calculation in GaSb/InAs core-shell NW. Based on the calculated power factor *S*^2^*G* and thermal conductance, we calculate the optimal ZT value in InAs/GaSb core-shell NW with different proportions at roon temperature as shown in Fig. 3(c). It is clearly shown that the 3*A*/3*B* has the maximum *ZT* value, since the 3*A*/3*B* has the lowest thermal conductance. Although the power factor of 3*A*/3*B* is lower than that of 6*A*/0*B*, the maximum *ZT* value originates from the competition between power factor and thermal conductance.

It is important to note that the shape of the *ZT* curve of InAs/GaSb NW is different from that of GaSb/InAs NW. This is due to the difference of phonon thermal conductance, which originates from the difference of thermal conductance between core and shell^28^. If the thermal conductance of core is larger than that of shell, the core is dominant thermal transport channel, however, in case the thermal conductance of core is less than that of shell, the dominant thermal transport channel will change from core to shell with the increasing shell thickness. This result also agrees with previous studies^28^,^30^. As shown in Fig. 2 and Fig. 3, the 5A/1B has the largest ZT due to the 5A/1B being of the lowest phonon thermal conductance, and so does 3B/3A. In short, the difference of *ZT* curve shape between GaSb/InAs core-shell NW and InAs/GaSb core-shell NW originates from the difference of thermal conductance in core and shell. However, this does not violate the conclusion that the thermoelectric performance of core-shell NW is much better than the single component NW at room temperature.

**Figure 4:**
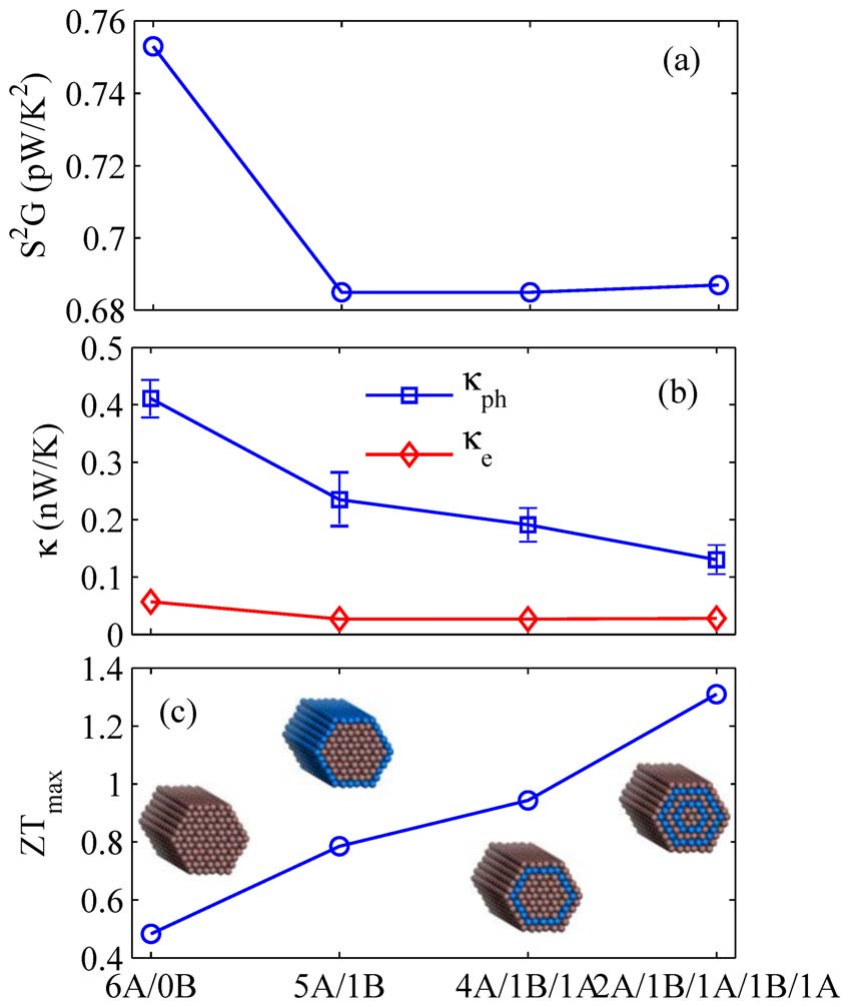
(a) Power factor *P*, (b) phonon thermal conductance *κ_ph_* and electron thermal conductance *κ_e_*, and (c) the optimal *ZT* in different core-shell NWs at room temperature, in comparison with those of GaSb NWs.

In order to further improve the thermoelectric performance of core-shell NWs exploratory, we design a novel multiple GaSb/InAs core-shell structure, and calculate the thermoelectric properties as shown in Figs. 4(a)–(c). As a comparison, the thermoelectric properties of GaSb NW are also given. Fig. 4(a) shows the power factor of the four different structures at room temperature. It is clearly shown that the core-shell NWs have lower power factor than the single component GaSb NWs, and the power factor is almost constant with the increasing shell number. This is due to the fact that the Seebeck coefficient and electrical conductance depend on the electrical band structure, and the electrical band structure of multiple core-shell structure is of little change with the increase of shell number, as shown in Fig. 1(b) and Fig. 1(d). The electron thermal conductance has a same trend as power factor due to the Wiedemann-Franz law, as shown in Fig. 4(b). In addition, the phonon thermal conductance rapidly goes down as the increasing shell number due to the enhancement of phonon-boundary scattering, which is very important in the quasi-one-dimensional nanowire due to the size effect and high surface to volume ratio^29^. In short, the decreasing phonon thermal conductance combining with the almost constant power factor leads to the increase of *ZT* in multiple core-shell NWs, as shown in Fig. 4(c). The *ZT* value of 2*A*/1*B*/1*A*/1*B*/1*A* can reach about three times greater than that of the single component GaSb NW at 300 K. In other words, the *ZT* in multiple core-shell NW can be enhanced significantly compared with the single component NW at room temperature due to the decreasing phonon thermal conductance and unaffected power factor.

**Figure 5:**
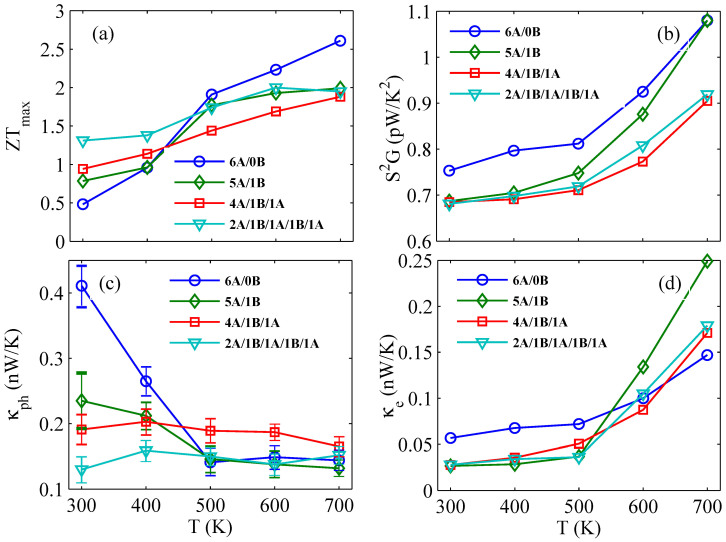
Temperature dependence of (a) *ZT*, (b) power factor *S*^2^*G*, (c) phonon thermal conductance *κ_ph_* and (d) electron thermal conductance *κ_e_* of different core-shell NWs, in comparison with those of GaSb NWs.

So far, we have seen that the thermoelectric performance of multiple core-shell NW is much better than that of single component NW at room temperature. In order to verify whether this advantage still exists at other temperatures, we plot the temperature dependence of *ZT* of different core-shell NWs, as shown in Fig. 5(a). From the panel (a), two facts are apparent: (i) The *ZT* values of the four structures are all increased as the temperature increases. (ii) The GaSb NW has a lower *ZT* value than that of other core-shell NWs at 300 K, but it goes up much faster than that of other core-shell NWs with increasing temperature, thus the *ZT* values of GaSb NWs exceed those of core-shell NWs in the temperature region above 400 K. In order to explain the two facts, we plot the power factor, phonon thermal conductance and electron thermal conductance of the four different structures as a function of temperature in Figs. 5(b)–(d), respectively. Obviously, the power factors of the four structures are all increased with the increasing temperature. It is because that a growing number of electrons can be excited into the conductance band and contribute to the electrical conductance with the increasing temperature. For the same reason, the electron thermal conductance has also a similar trend with the power factor due to the charge carriers are also heat carriers. Besides, the phonon thermal conductance of the GaSb NWs rapidly goes down as the temperature increases due to the significant enhancement of Umklapp phonon-phonon scattering. On the contrary, the phonon thermal conductance of core-shell NWs shows little change with the increasing temperature. Therefore, the increasing power factor and electron thermal conductance combining with the decreasing phonon thermal conductance lead to the increase of *ZT* values as the temperature is increased. In addition, the rapid increase of *ZT* value in GaSb NW is because the phonon thermal conductance goes down rapidly due to the enhancement of Umklapp phonon-phonon scattering with the increasing temperature, and the slow increase of *ZT* value in core-shell NWs is due to the phonon thermal conductance shows little change with the increasing temperature as shown in Fig. 5(c). In short, the multiple core-shell NWs is more suitable as thermoelectric material at room temperature, but the single component NWs is more suitable as thermoelectric material at high temperature.

The temperature dependence of thermal conductance in core-shell NWs is shown in Fig. 5(c). The phonon thermal conductance of the GaSb NWs rapidly goes down as the temperature increases, but that of core-shell NWs is of little change. The similar phenomenon also appears in the Si/Ge core-shell NW^30^. This phenomenon can be understood from the phonon scattering mechanism. The increase of the temperature has two effects on phonon thermal conductance. On the one hand, the Umklapp phonon-phonon scattering is enhanced with an increase in temperature. On the other hand, with the increase of temperature, the heat capacities of phonons increase significantly, especially for the high-frequency phonons^29^. So the temperature dependence of thermal conductance is determined by the competition between the two effects.

## Discussion

In summary, the thermoelectric properties of InAs and GaSb multiple core-shell NWs are investigated by using nonequilibrium Green's function method and molecular dynamics simulations. A slight decrease of power factor is observed when the core-shell structure is introduced, associated with remarkable reduction of phonon thermal conductance. These two forces make the *ZT* of core-shell NWs increase obviously. We also find that the thermoelectric performance of multiple core-shell NWs is significantly improved due to the remarkable reduction of phonon thermal conductance. The *ZT* value can reach three times greater than that of the single component GaSb NW at room temperature. Moreover, we investigate the influence of temperature on the thermoelectric properties in core-shell NWs. The result shows that the multiple core-shell NW is more suitable as thermoelectric material at room temperature, but the single component NW is more suitable as thermoelectric material at high temperature.

## Methods

We adopt the nonequilibrium Green's function method in combination with density functional theory, which is implemented in the Atomistix ToolKit (ATK) software package^31^,^32^, to optimize structure and calculate the electrical transmission. Here, a ballistic transport is assumed, and this will not affect the relative variation of the electrical conductance in different structures. The single-zeta plus polarization basis set are employed, and the exchange-correlation potential is described by the local-density approximation. The cutoff energy is set to 150 Ry. The Brillouin zone is sampled with 1 × 1 × 100 Monkhorst-Pack k-mesh. All atomic positions are relaxed until the maximum atomic force becomes smaller than 0.02 eV/A. According to the Caroli formula, the electron transmission is calculated as 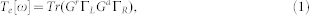
where *G^r^* = (*G^a^*)^†^ is the advanced Green function of the central scattering region and Γ*_L_*_(*R*)_ represents the coupling interaction with the left (right) semi-infinite lead. Thus, the electrical physical quantities involved in the *ZT* formula can be calculated by, respectively, 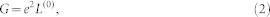

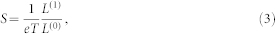

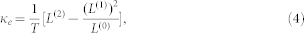
With 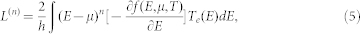
where *f*(*E*,*μ*,*T*) is the Fermi-Dirac distribution function at the chemical potential *μ* and temperature *T*.

We calculate the lattice thermal conductance by using the nonequilibrium molecular dynamics (NEMD) simulations implemented in the LAMMPS software package. In our simulations, the Tersoff potential, which is widely used in the atomistic simulations of the group III–V semiconductors, is employed to model the atomic interactions^33^,^34^,^35^,^36^,^37^. The parameterization of the Tersoff potential we used, which is obtained by fitting the 12 parameters in the functional form to experimental and density functional theory calculated values of the cohesive energy, the lattice parameter, the three elastic constants, comes from the reference 34. The initial structure is relaxed in Nosé-Hoover thermostats at temperature T, T is the average temperature. The velocity Verlet algorithm is used to integrate the differential equations of motions with an integration time step of 1.0 fs. After structure relaxation for 1 × 10^6^ steps, the Nosé-Hoover heat baths with different temperaetures, *T_L_* = *T* + Δ*T* and *T_R_* = *T* − Δ*T*, are applied to the two ends of nanowires to establish a temperature gradient along the longitudinal direction. The simulations are performed long enough (2 × 10^7^ time steps) such that the system reaches a nonequilibrium stationary state where the heat current going through the system is time independent, the thermal conductivity is calculated from the Fourier law, *λ_ph_* = *J*/∇*T*, where *J* is the heat flux along the longitudinal direction, and ∇*T* is the temperature gradient, which is calculated from linear fitting to the temperature profile, and it can be written as ∇*T* = (*T_L_* − *T_R_*)/*L*, with *L* is the length of NWs. Having obtained the *λ_ph_*, the thermal conductance can be calculated by *κ_ph_* = *λ_ph_*·*S*/*L*, where *S* is the cross-sectional area. In addition, the length of all the nanowires in our simulation is about 20 nanometres. It is worth noting that the NEMD results heavily depend on the system size. However, the focus of the work is the effect of core-shell structure for thermoelectric properties in the same system size, so the finite-size effects will not affect the conclusions.

## Author Contributions

K.Q.C. and W.X.Z. conceived the research. W.X.Z. did the numerical simulations. K.Q.C. and W.X.Z. oversaw all research phases. Everyone contributed to the writing of the paper.
